# Supplementary material for: Iron (II) Metallo-Supramolecular Polymers Based on Thieno[3,2-b]thiophene for Electrochromic Applications
Source: Polymers (Basel). 2021 Jan 23;13(3):362. doi: 10.3390/polym13030362 (PMC7865520; doi:10.3390/polym13030362)
Supplement: Supplementary file 1 [file polymers-13-00362-s001.pdf]

Iron (II) metallo-supramolecular polymers based on thieno[3,2-*b*]thiophene for electrochromic applications

Andrei Chernyshev,[1,#] Udit Acharya,[2,3,#] Jiří Pfleger,[2] Olga Trhlíková,[2] Jiří Zedník[1] and Jiří Vohlídal[1*]

[1] Charles University, Faculty of Science, Department of Physical and Macromolecular Chemistry, Hlavova 2030, 128 40 Prague 2, Czech Republic;

[2] Institute of Macromolecular Chemistry, Academy of Sciences of the Czech Republic, Heyrovského nám. 2, 162 06, Prague, Czech Republic;

[3] Faculty of Mathematics and Physics, Charles University, 121 16 Prague 2, Czech Republic;

[#] Both co-authors contributed equally to this work.

* Correspondence author: Jiří Vohlídal[1] , E-mail: vohlidal@natur.cuni.cz;

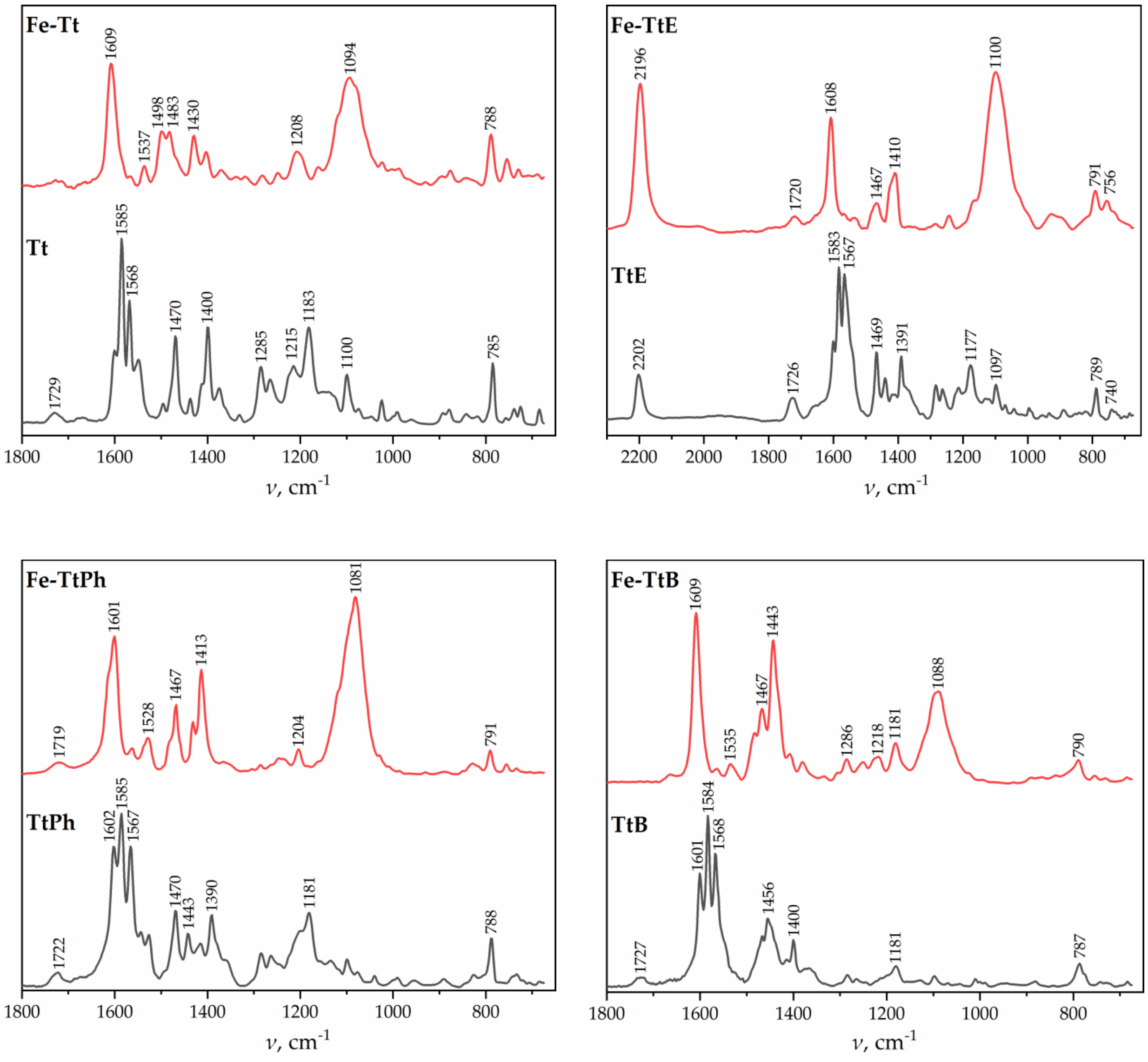

Figure S1. IR spectra of unimers and corresponding Fe-MSPs.

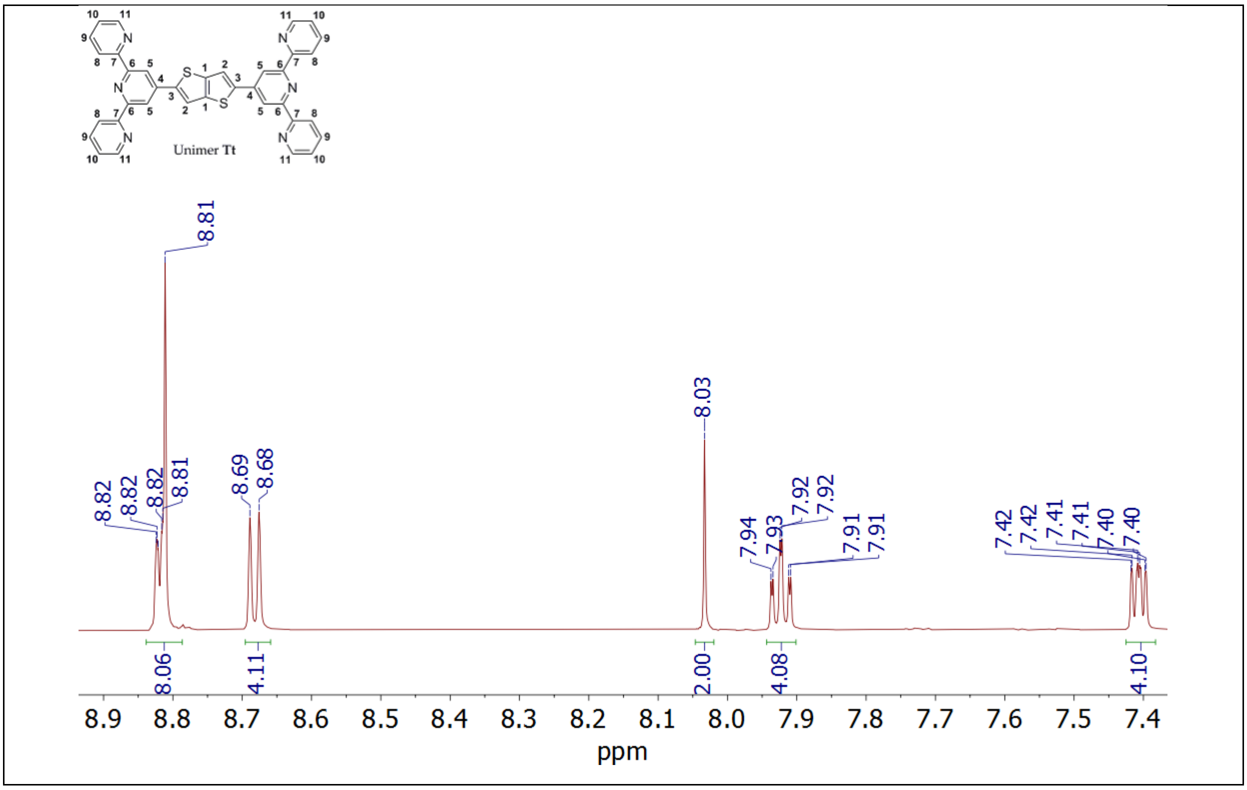

Figure S2. ^{1}H NMR spectra of unimer **Tt**.

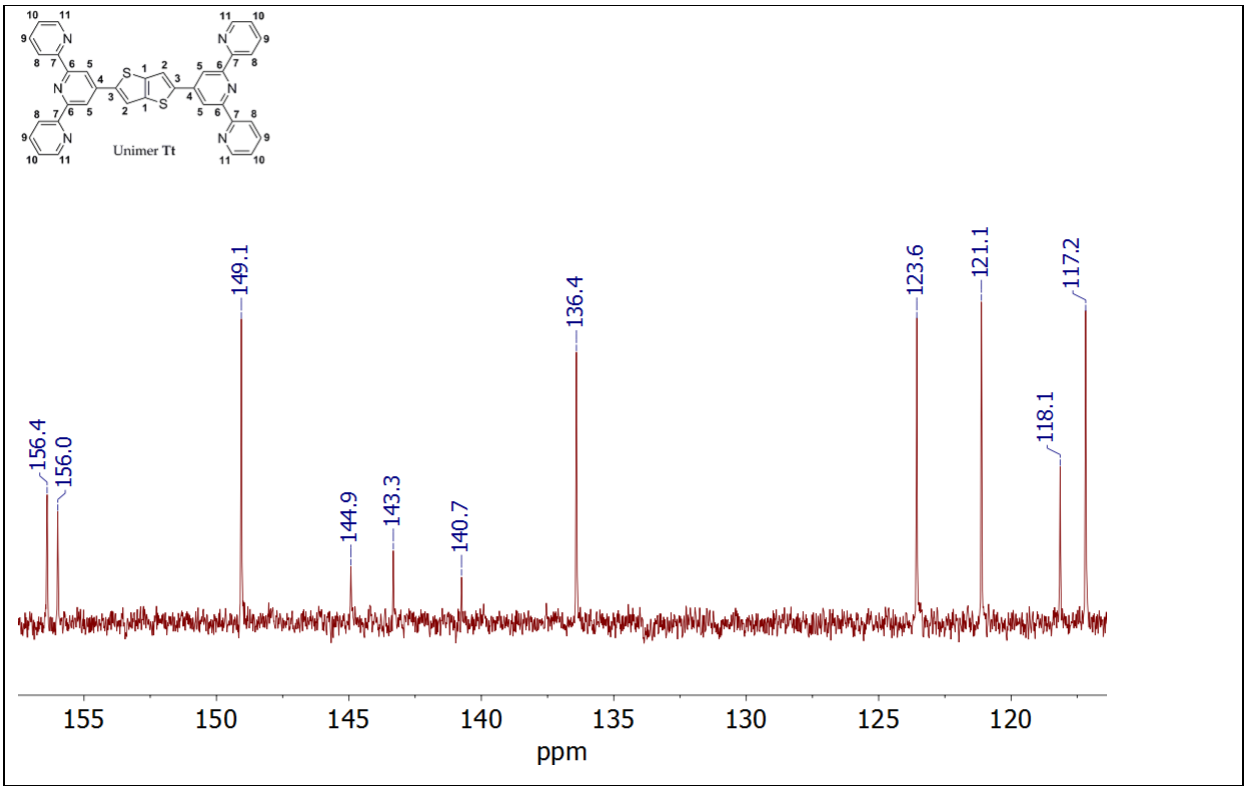

Figure S3. ^{13}C NMR spectra of unimer **Tt**.

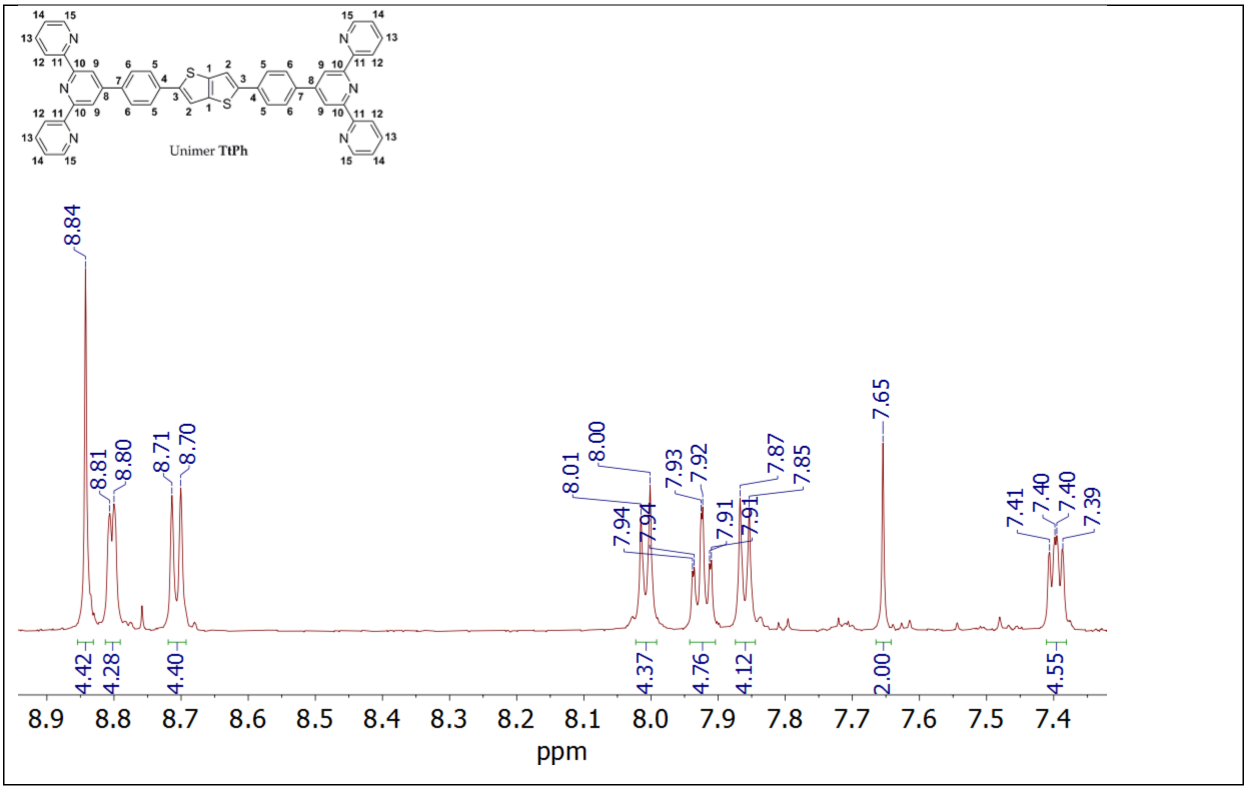

Figure S4. ^{1}H NMR spectra of unimer **TtPh**.

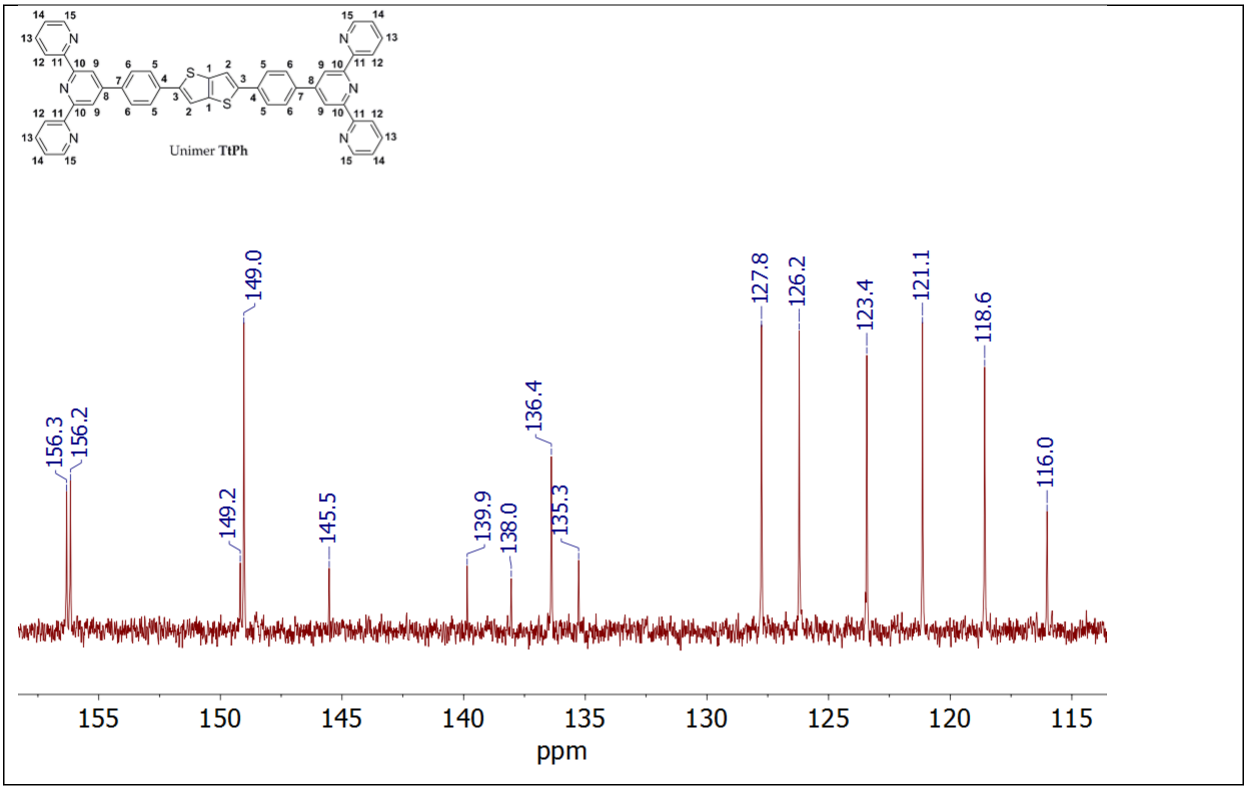

Figure S5. ^{13}C NMR spectra of unimer **TtPh**.

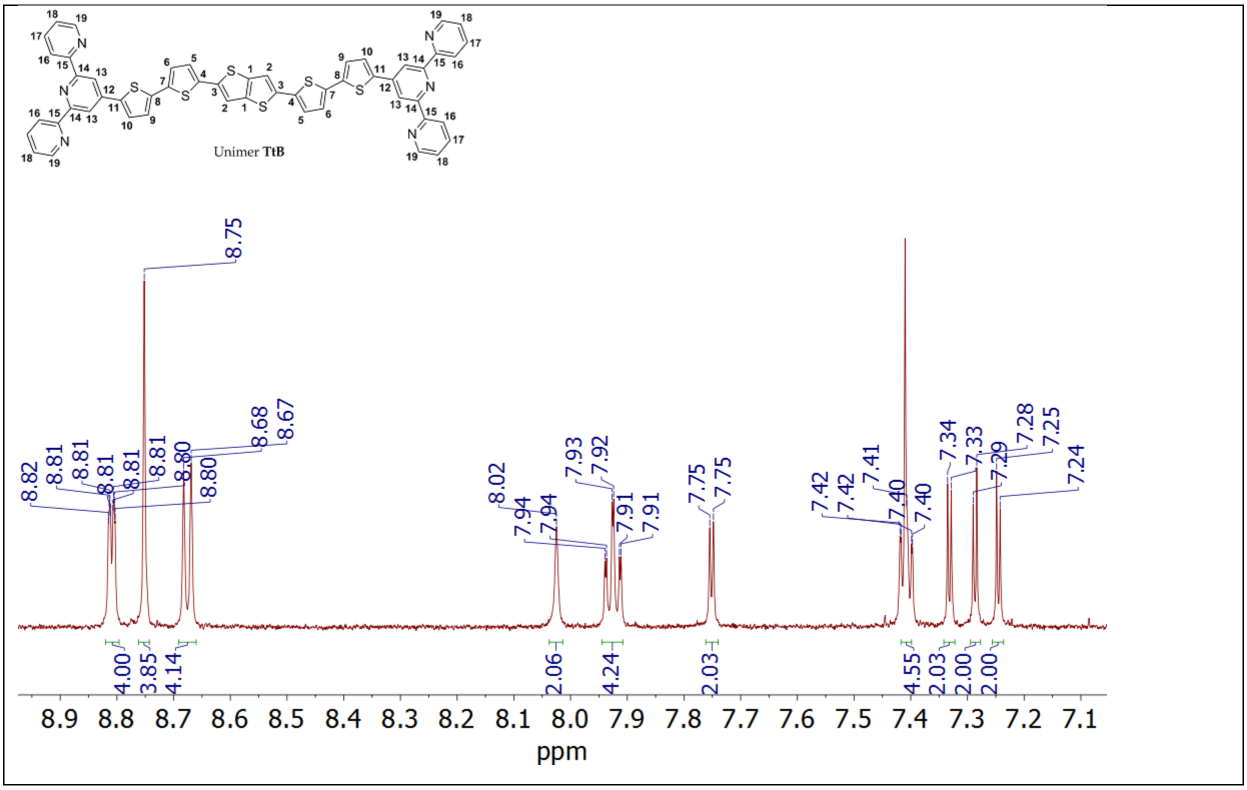

Figure S6. ^{1}H NMR spectra of unimer **TtB**.

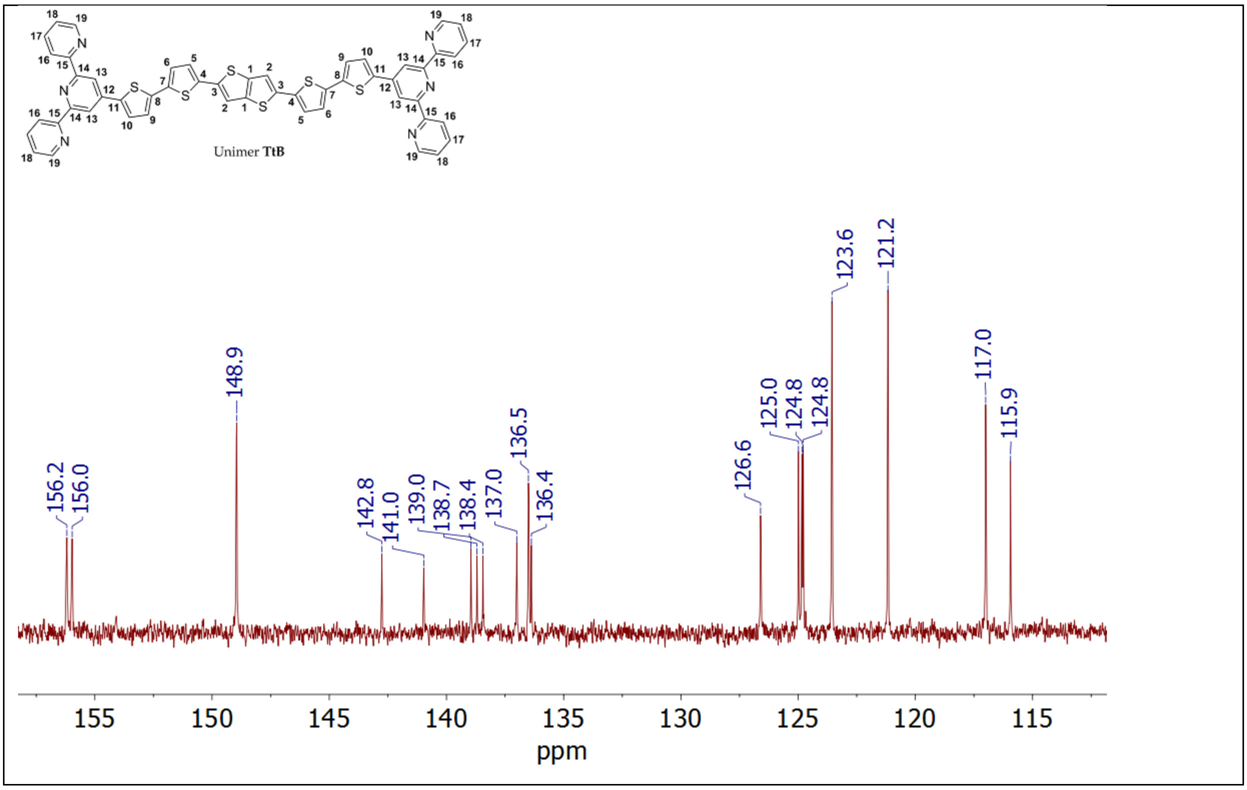

Figure S7. ^{13}C NMR spectra of unimer **TtB**.

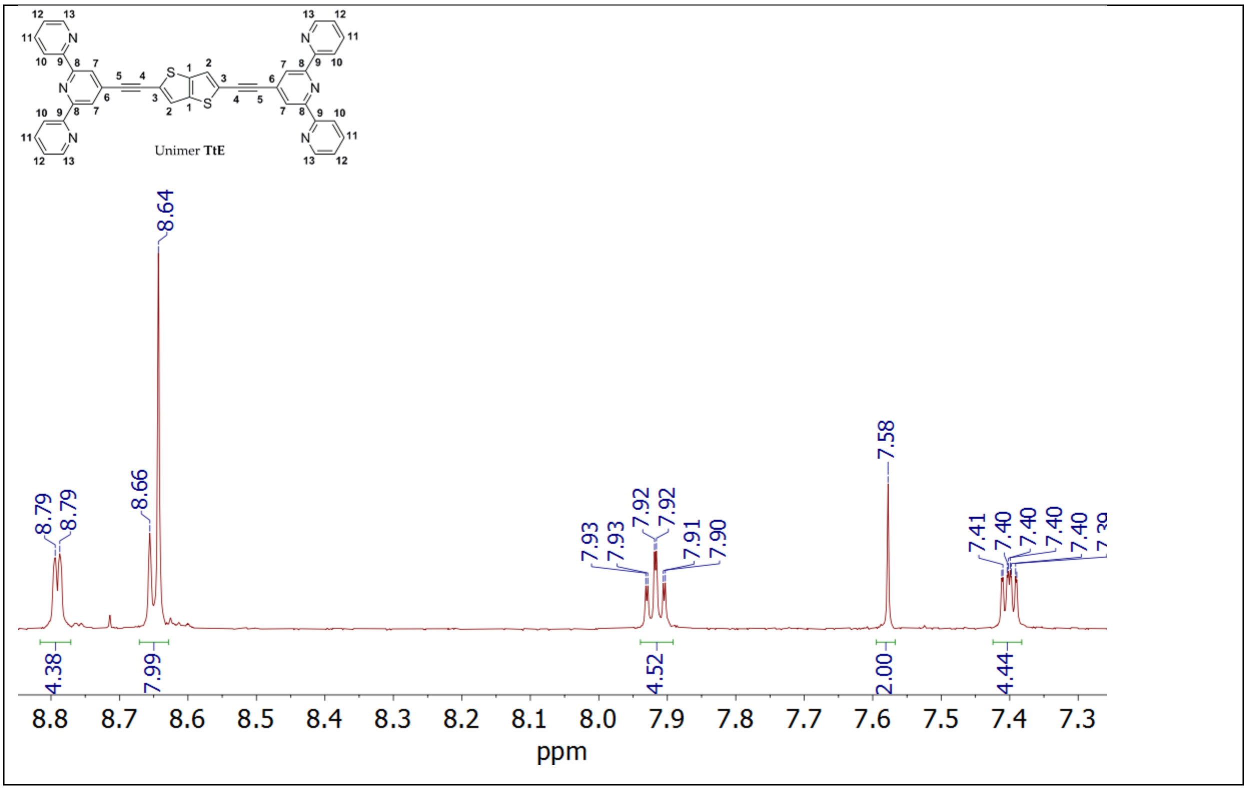

Figure S8. ^{1}H NMR spectra of unimer **TtE**.

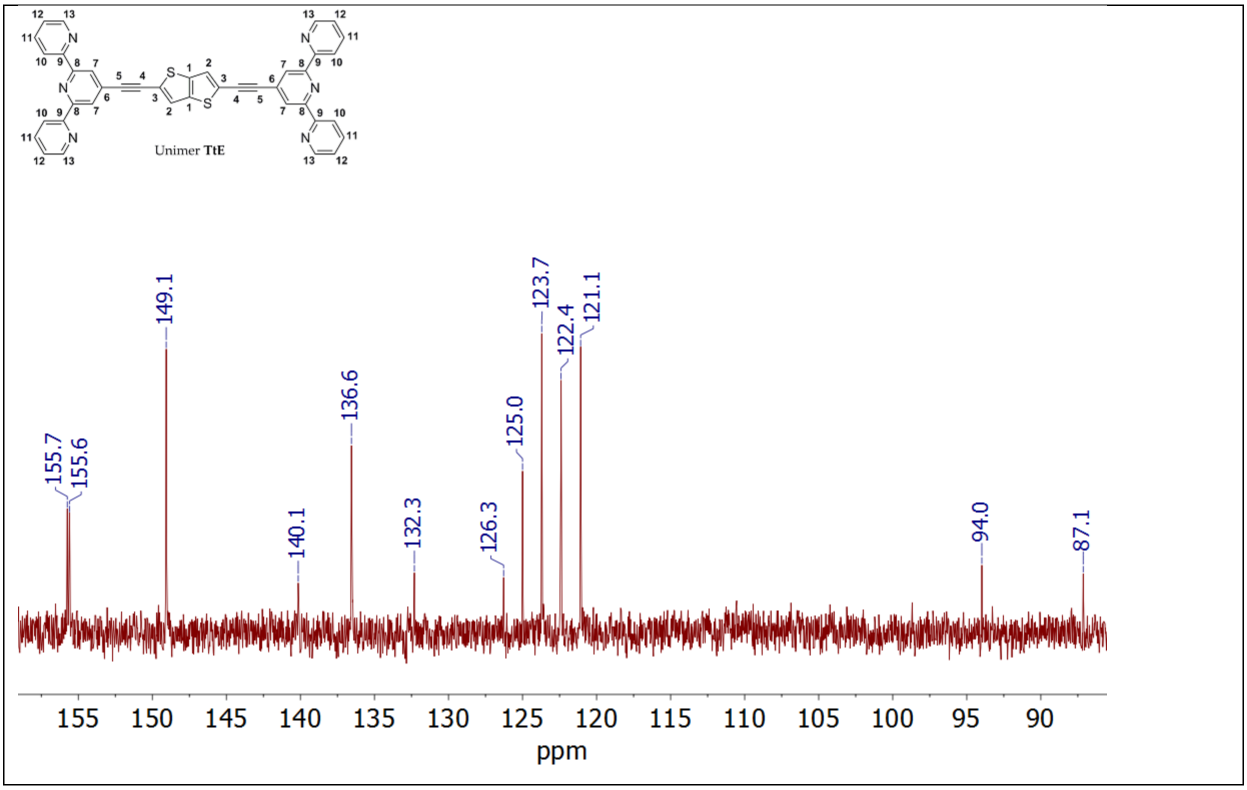

Figure S9. ^{13}C NMR spectra of unimer **TtE**.

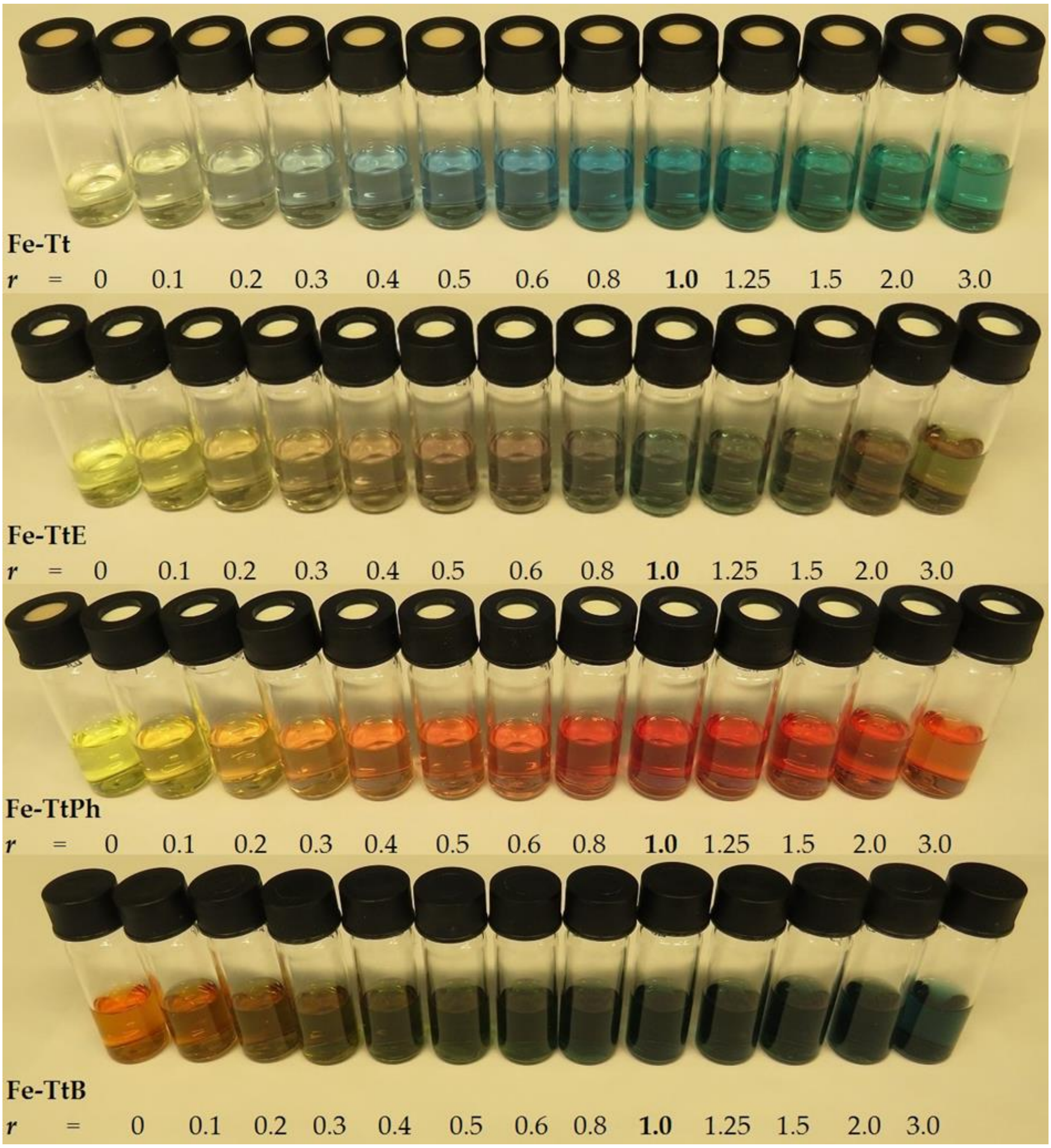

Figure S10. Solutions with gradually increasing ratio r = [Fe^{2+}]/[U] clearly show the effect of linker on the color of metallo-supramlecular oligomers and polymers.

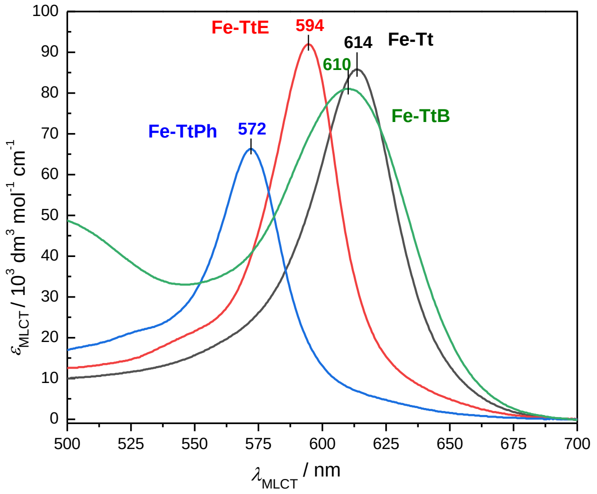

Figure S11. Comparison of the position and intensity of MLCT bands of Fe-MSPs (r = 1) solutions.

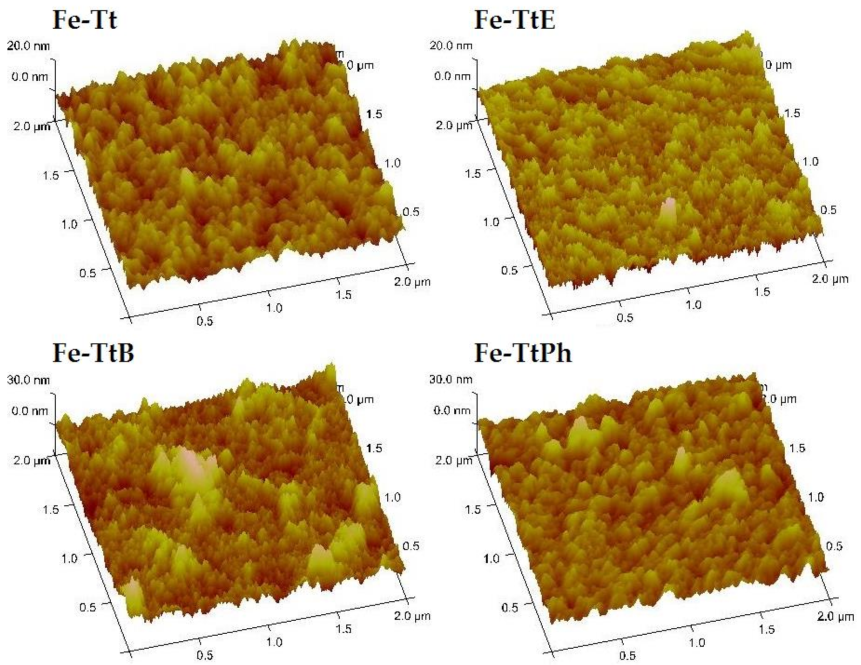

Figure S12. AFM images of Fe^{2+}-MSP films (tapping mode, clockwise).

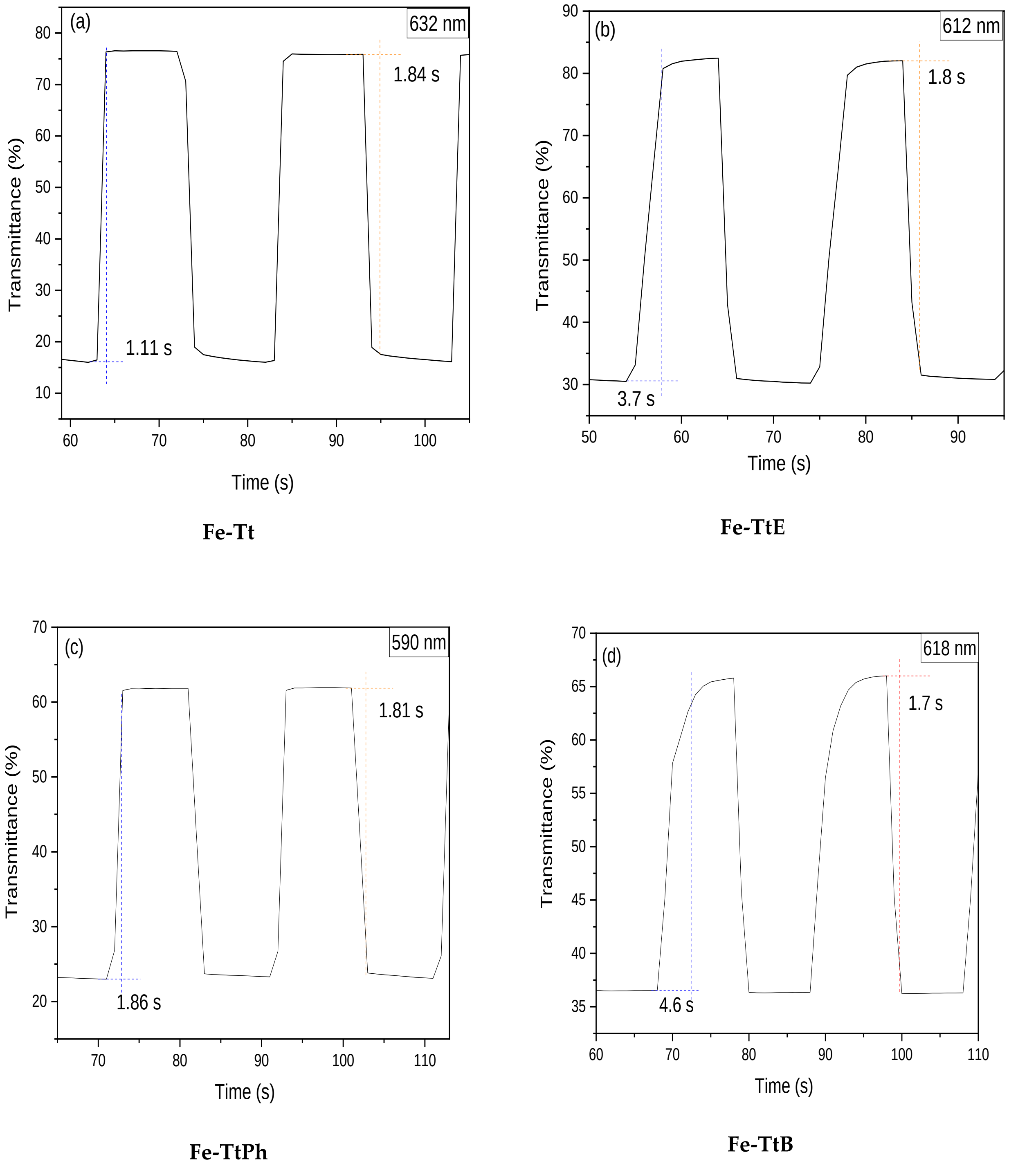

Figure S13. Determination of the bleaching and coloring time of electrochromic films from 95 % of saturated transmission. (a) **Fe-Tt;** (b) **Fe-TtE;** (c) **Fe-TtPh**; and (d) **Fe-TtB.**

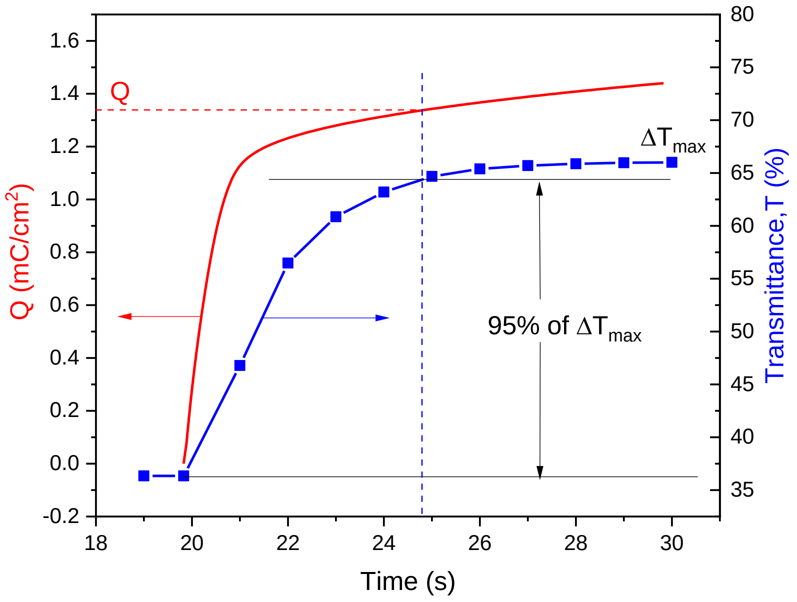

Figure S14. Time courses of the transmittance, T, at $\lambda = 618$ nm of the **Fe-TtB** electrochromic layer and the charge passed through the layer, Q, during its bleaching. The charge needed to achieve 95% of the final saturated transmittance was used for the calculation of the coloration efficiency

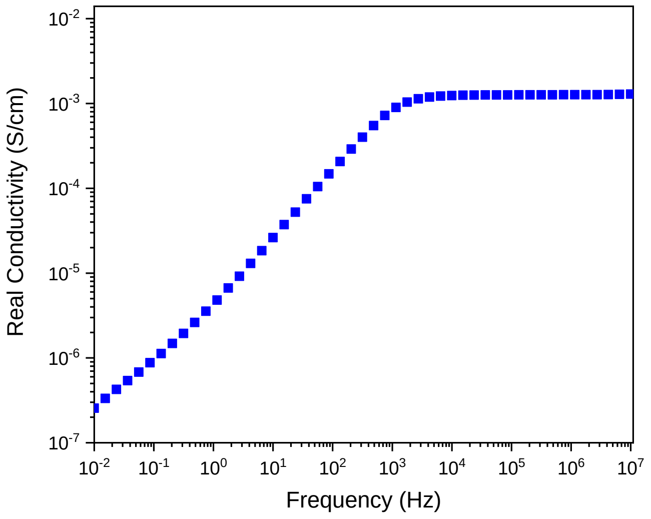

Figure S15. Frequency dependence of the real part of the conductivity of gel electrolyte.

Randles-Ševčik equation [35,36] for temperature of 25 °C is:

$$i_p = 269 \cdot c \cdot D^{1/2} \cdot v^{1/2}$$

where: i_p is the current density of CV peak, c the molar concentration (in mol/L) and D diffusion coefficient (in cm^2s^{-1}) of electroactive species and v the scan rate (in $V \cdot s^{-1}$).
